# *Helicobacter pylori* Infection following Endoscopic Resection of Early Gastric Cancer

**DOI:** 10.1155/2019/9824964

**Published:** 2019-10-16

**Authors:** Lan Li, Chaohui Yu

**Affiliations:** Department of Gastroenterology, The First Affiliated Hospital, College of Medicine, Zhejiang University, Hangzhou, China

## Abstract

The role of *Helicobacter pylori* (*H. pylori*) infection in patients following endoscopic resection of early gastric cancer (EGC) remains unclear. This article presents a review of literature published in the past 15 years. *H. pylori*‐mediated persistent methylation levels are associated with the development of metachronous gastric cancer. The methylation of certain specific genes can be used to identify patients with a high risk of metachronous gastric cancer even after* H. pylori* eradication. *H. pylori* eradication after endoscopic resection should be performed as early as possible for eradication success and prevention of metachronous precancerous lesions. Although whether the eradication of *H. pylori* could prevent the development of metachronous cancer after endoscopic resection is controversial, several meta‐analyses concluded that *H. pylori* eradication could reduce the incidence of metachronous gastric cancer significantly. In addition, *H. pylori* eradication in gastric cancer survivors after endoscopic resection could reduce healthcare cost and save lives in a cost‐effective way. Taken together, *H. pylori *eradication after endoscopic resection of EGC is recommended as prevention for metachronous precancerous lesions and metachronous gastric cancer.

## 1. Introduction

In recent years, endoscopic resection including endoscopic mucosal resection (EMR) and endoscopic submucosal dissection (ESD) has been widely used in the treatment of early gastric cancer (EGC) [1]. The risk of the development of metachronous gastric cancer in patients who underwent endoscopic resection of EGC is higher than that in gastrectomized patients. Endoscopic resection is minimally invasive and preserves the whole stomach, which could increase the risk of metachronous cancers on unresected parts of the stomach compared to surgical resection [2, 3]. *Helicobacter pylori* (*H. pylori*) is the major cause of gastric carcinogenesis, and its infection leads to chronic gastritis, atrophic gastritis, intestinal metaplasia, dysplasia, and subsequently to gastric adenocarcinoma [4, 5]. The role of *H. pylori* infection in patients following endoscopic resection of EGC remains unclear due to different results from various studies. It is still controversial whether the eradication of *H. pylori* can reduce the likelihood of metachronous gastric cancer. In the present study, we will review the literature on the relationship between *H. pylori* infection and metachronous gastric lesion after endoscopic resection. The purpose of the present study is to explore the role of *H. pylori* infection and *H. pylori* eradication in the patients after endoscopic resection of EGC.

## 2. Prevalence of *H. pylori* Infection after Endoscopic Resection of Early Gastric Cancer

Endoscopic therapy is a minimally invasive treatment that allows the patient to preserve the entire stomach. The anatomy of the stomach has no significant change after endoscopic resection for EGC, but few studies have examined whether the microbiological profile in the stomach has changed. Prevalence of *H. pylori* infection after endoscopic resection for EGC has seldom been reported. Hwang et al. reported that* H. pylori *infection rate after endoscopic resection of EGC was significantly higher in the patients with residual tumors group than in the patients without residual tumors group [6]. Moreover, *H. pylori *were independent factors of the presence of residual tumor in additional gastrectomy after incomplete endoscopic resection for EGC [6]. Large‐scale studies are needed to determine whether there is a difference in *H. pylori* infection rate between general population and the patients following endoscopic surgery of EGC.

## 3. Effect of *H. pylori* on Ulcers Developing after Endoscopic Resection of Early Gastric Cancer

Several studies have examined the effect of *H. pylori* eradication on ulcers developing after endoscopic resection of EGC. Song et al. suggested that early *H. pylori* eradication therapy can promote *H. pylori*-positive ESD‐induced artificial ulcer healing [7]. Cheon et al. found that *H. pylori* eradication was better than PPI treatment with respect to the healing of artificial gastric ulcer after EMR for EGC [8]. It was reported that *H. pylori* infection led to decreased mucosal blood flow at the margin of EMR‐induced ulcers, which indirectly supports the above report [9]. However, in one study concerning the effect of *H. pylori* infection status on post‐EMR gastric ulcers by Kakushima et al. [10], the healing rate of ulcers at 8 weeks after EMR was not affected by *H. pylori* infection status. Likewise, several reports have shown that the infection status of *H. pylori* does not affect ulcer healing after ESD [10–14]. The reasons for these are as follows: Eradication therapy promotes the healing process of peptic ulcers by improving microcirculation. However, muscular contraction might be a major factor in the healing process of artificial ulcers, while improvement of microcirculation is only a secondary factor. In addition, the pathogenesis of artificial ulcers is completely mechanical rather than *H. pylori*‐induced apoptosis or gastric juice‐induced degradation. Further follow‐up prospective studies will help to clarify the effect of *H. pylori *on ulcers developing after endoscopic resection of EGC.

## 4. Molecular Pathogenesis of *H. pylori*‐Related Metachronous Gastric Cancer

**Figure 1:**
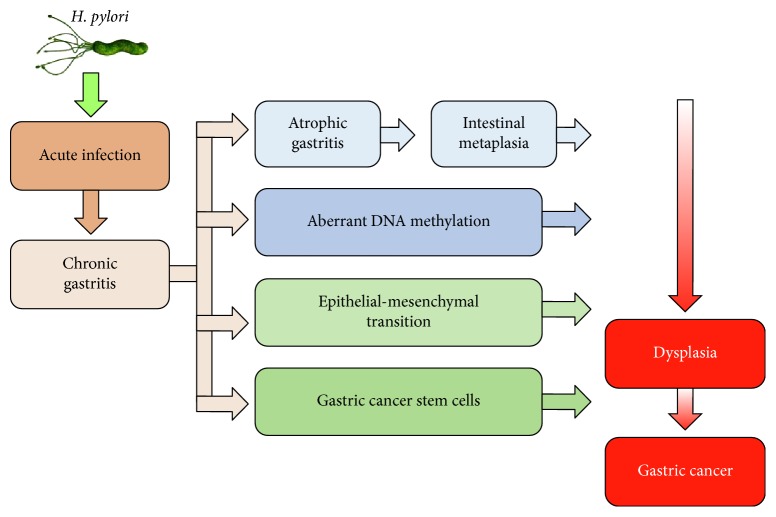
A model representing the role of *H. pylori* in the development of gastric cancer.

The current studies found that the mechanisms by which* H. pylori* infection might eventually lead to gastric cancer included *H. pylori*‐induced atrophic gastritis and intestinal metaplasia, aberrant DNA methylation, epithelial–mesenchymal transition, and gastric cancer stem cells (Figure 1) [15]. Chronic active *H. pylori* infection leads to the recruitment of immune cells, increasing secretion of interleukin‐1*β*, tumor necrosis factor‐*α*, and reactive oxygen species, which together cause the activation of DNA methyltransferase 1, mediating abnormal DNA methylation in the gastric mucosa [16]. The methylation level in gastric mucosa is partially reversible by *H. pylori* eradication in a gene‐specific manner [17–19], but the methylation of some molecules in stem cells can last for a long time [20]. Thus, it is likely that the methylation level after *H. pylori* eradication could reflect the epigenomic damage of stem cells [20, 21]. It was reported that the methylation of MOS and miR‐124a‐3 could predict the risk of metachronous gastric cancer [20, 22]. The methylation level of MOS in patients with metachronous gastric cancer was significantly higher than that in patients without metachronous gastric cancer [22]. Likewise, high miR‐124a‐3 methylation level was correlated with an increased risk of developing metachronous gastric cancers [20]. Therefore, *H. pylori*‐mediated persistent methylation levels are associated with the development of metachronous gastric cancer. The methylation of certain specific genes can be used to identify patients with a high risk of metachronous gastric cancer even after *H. pylori* eradication.

## 5. Effect of *H. pylori* on Metachronous Precancerous Lesions after Endoscopic Resection of Early Gastric Cancer

Many studies reported the results concerning the effect of *H. pylori* on gastric atrophy and intestinal metaplasia following endoscopic resection of EGC. Choi et al. [23] and Han et al. [24] reported that the grade of atrophy on corpus was significantly lower in the *H. pylori*‐eradicated group than in the persistent group during a follow‐up period longer than 5 years after endoscopic treatment of EGC. Zhang et al. found that *H. pylori* infection was an independent risk factor for recurrence of gastric mucosal dysplasia after endoscopic resection [25]. Similarly, the multivariate analysis by Chon et al. showed that eradication of *H. pylori* was related to reduce incidence of subsequent gastric dysplasia [26]. Of particular significance is the observation that failure of *H. pylori* eradication occurred more frequently in metachronous patients with gastric dysplasia than in those with carcinoma [27]. In other words, eradication failure was closely related to dysplasia, but not carcinoma, in the metachronous group. Therefore, *H. pylori* infection could play a role in the development of metachronous precancerous lesions after endoscopic resection of EGC. There may be a ‘point of no return,' beyond which molecular changes are irreversible and *H. pylori* eradication could no longer prevent metachronous lesions. It is difficult to correctly define the point of no return because the molecular process cannot be determined precisely. On the basis of the above studies, *H. pylori* eradication after endoscopic resection of EGC should be performed as early as possible for the prevention of metachronous precancerous lesions.

## 6. Effect of *H. pylori* Eradication on the Development of Metachronous Gastric Cancer

Although *H. pylori* is a well‐known risk factor and plays an important role in the development of gastric cancer, the effect of *H. pylori* infection on the development of metachronous gastric cancer after endoscopic resection of EGC still remains unclear [28]. The mucosa adjacent to *H. pylori*‐infected gastric cancer is usually accompanied by atrophy and intestinal metaplasia, which is susceptible to develop metachronous gastric cancer. Endoscopic resection preserves the gastric mucosa to the greatest extent, so the incidence of metachronous gastric cancer in the abnormal background mucosa is significantly increased [2]. Previous studies illustrated that persistent *H. pylori* infection was associated with an increased risk of subsequent gastric dysplasia or cancer after endoscopic resection of EGC, and *H. pylori* infection is an independent risk factors for metachronous gastric cancer after ESD of EGC [29–31]. However, Lim et al. showed an inverse relationship between *H. pylori* infection and metachronous gastric cancer [32]. One possible reason is that *H. pylori*‐negative patients may have previously been infected with *H. pylori* and previous long‐term infection might have a greater effect on the development of metachronous gastric cancer than newly developed current infection.

It is still on debate whether the eradication of *H. pylori* could prevent the development of metachronous cancer after endoscopic resection of EGC. Several long follow‐up prospective studies reported that eradication of *H. pylori* in the patients treated with endoscopic resection of gastric dysplasia or EGC could reduce the risk of metachronous cancer [23, 33, 34]. However, a 3‐year prospective study by Choi et al. reported that the cumulative incidence of metachronous gastric cancer was not different between patients with or without *H. pylori* eradication [35]. Other retrospective studies showed similarly controversial conclusions [26, 27, 31, 36–40]. The reasons for this discrepancy are as follows: First, the study designs were different among the studies. Some were prospective randomized trials, and others were retrospective cohort studies. Second, the timing of the *H. pylori* eradication was not consistent. Some persons received eradication treatment immediately after endoscopic resection, and others received treatment several months after endoscopic resection. Third, the follow‐up time was not consistent among the studies and most studies had a follow‐up period of less than 5 years. In addition, the methods for calculations of the observation period were different among the studies. Some started from eradication of *H. pylori*, and others started from the initial ESD.

Nevertheless, several meta‐analyses concluded that *H. pylori* eradication could reduce the incidence of metachronous gastric cancer significantly, therefore preventing against metachronous gastric cancer in patients who have undergone endoscopic resection [41–44]. Although it is possible to develop metachronous gastric cancer after successful eradication of* H. pylori*, *H. pylori* eradication after endoscopic resection of EGC is still recommended in the Maastricht consensus report and the Guidelines of the Japanese Gastroenterological Society [45, 46]. Further prospective studies with long‐term follow‐up could help clarify the effect of *H. pylori* eradication in preventing metachronous gastric cancer after endoscopic resection of EGC.

Furthermore, Park et al. clarified that *H. pylori* infection or eradication did not affect the time interval between the initial ESD and the development of metachronous gastric cancer [47], which implied that *H. pylori* eradication might not halt the progression of precancerous lesions, especially when the neoplastic change has reached a certain degree. A possible reason is that metachronous gastric lesions might have already been in the stomach at the time of ESD for EGC as tiny or invisible lesions, and therefore, the time interval to occurrence of metachronous gastric cancer might not be affected by *H. pylori* eradication in patients with undetected early cancer or slowly growing latent cancer [48].

## 7. Optimal Timing for *H. pylori* Eradication for Patients after Endoscopic Resection of Early Gastric Cancer

Few studies have explored the optimal timing to eradicate *H. pylori* after endoscopic resection of EGC. Huh et al. assessed whether the eradication of *H. pylori* at different time points after endoscopic resection affected the success rates of eradication therapy [49]. They concluded that the early treatment group (≤2 weeks after endoscopic resection) achieved a significantly higher eradication rate than the intermediate group (2–8 weeks) or the late group (≥8 weeks). Multivariate analysis showed that early initiation of *H. pylori* eradication therapy was an independent significant predictor of eradication success. On the other hand, it was reported that *H. pylori* eradication in the patients who underwent ESD for EGC should be performed before the progression of gastric mucosal atrophy [36]. The presence of moderate‐to‐severe mucosal atrophy or intestinal metaplasia may be a ‘point of no return' and does not appear to regress following *H. pylori* eradication [36, 50]. These results suggest that eradication of *H. pylori* as early as possible after endoscopic surgery offers the greatest chance of eradication success.

## 8. Cost‐Effectiveness of *H. pylori* Eradication in Patients after Endoscopic Resection of Early Gastric Cancer

Patients after endoscopic resection of EGC are more susceptible to metachronous gastric cancer and might benefit more from *H. pylori* eradication. Shin et al. reported that the health care cost for patients after endoscopic resection of EGC was significantly lower in *H. pylori* eradication group than in no eradication group (US$29,780 vs. US$30,594), while mean life expectancy from eradication was significantly longer in *H. pylori* eradication group (13.60 years vs. 13.55 years) [51]. Therefore, eradication of *H. pylori* in gastric cancer survivors after endoscopic resection could reduce healthcare costs while saving lives, and these findings have favorable implication in decision making about the most appropriate treatment option after endoscopic resection of EGC. Large‐scale studies are needed to clarify whether this strategy could be cost‐effective in selective population with high risk of developing metachronous gastric cancer.

## 9. Conclusion

In this review, we summarized the studies addressing *H. pylori *infection after endoscopic resection of EGC. Unfortunately, prevalence of *H. pylori* infection after endoscopic resection of EGC has seldom been reported. The effect of *H. pylori* eradication on ulcers developing after endoscopic resection is still on debate.

*H. pylori*‐mediated persistent methylation levels are associated with the development of metachronous gastric cancer. The methylation of certain specific genes can be used to identify patients with a high risk of metachronous gastric cancer even after *H. pylori* eradication. *H. pylori* eradication after endoscopic resection should be performed as early as possible for eradication success and prevention of metachronous precancerous lesions. Although whether the eradication of *H. pylori* could prevent the development of metachronous cancer after endoscopic resection is controversial, several meta‐analyses concluded that *H. pylori* eradication could reduce the incidence of metachronous gastric cancer significantly. In addition, *H. pylori* eradication in gastric cancer survivors after endoscopic resection could reduce health care cost and save lives in a cost‐effective way. Taken together,* H. pylori* eradication after endoscopic resection of EGC is recommended as prevention for metachronous precancerous lesions and metachronous gastric cancer. A large‐scale, long‐term research is needed to examine the role of *H. pylori* infection and *H. pylori* eradication in the patients following endoscopic resection of EGC.
